# HIV transmission between spouses living in Lu'an city, Anhui province, China: a longitudinal study

**DOI:** 10.1017/S095026881900089X

**Published:** 2019-05-29

**Authors:** Gongyan Ma, Haiyan Chen, Jin Chen, Zhenghao Jiang, Tai Ma

**Affiliations:** 1 Lu'an Center for Disease Control and Prevention, Lu'an, China; 2 School of Basic Medical Science, Anhui Medical University, Hefei, China

**Keywords:** Discordant couples, HIV, longitudinal study

## Abstract

This study aims to investigate the human immunodeficiency virus (HIV) transmission rate in HIV serodiscordant couples, in addition to the relevant influencing factors. From January 1999 to August 2016, patients with HIV/AIDS (index cases) along with their fixed partners were registered and monitored to determine the rate of HIV transmission between couples, as well as relevant influencing factors. A total of 231 HIV-positive couples were investigated, of these, 45-negative (19.48%) partners were infected with HIV via sexual transmission prior to diagnosis of the first case detected in couples. After diagnosis, the transmission rate between spouses was 0.39 per 100 person-years (2/507.7), and the cumulative transmission rate was 1.08% (2/186), which was significantly lower than the transmission rate before diagnosis (*χ*^2^ = 35.714, *P* < 0.001). Among the 119 HIV/AIDS patients who received antiretroviral therapy (ART), the transmission rate was 0 (0/119), whereas the transmission rate was 2.99% (2/67) in HIV/AIDS patients who did not receive ART. In addition, HIV transmission rate in serodiscordant couples was high prior to diagnosis of the index case. However, following diagnosis, the transmission rate was reduced, and the risk of transmission in the index case with antiviral treatment was null. Therefore, a prompt intervention in HIV discordant couples with ART of index case is vital to reduce the risk of HIV transmission.

## Introduction

In China, there has been a fundamental change in the epidemic pattern of human immunodeficiency virus (HIV), mainly due to blood transmission in the mid-1990s to sexual transmission in recent years [1]. In 2016, a total of 115 465 new cases of HIV/AIDS have been reported in China, of which 76 492 (over 66%) were in heterosexuals [2]. Previous studies have shown that heterosexual transmission had become the most widespread method of HIV transmission in mainland China [2–4]. Heterosexual transmission consists of three types: commercial sexual partners, spouses or fixed sexual partners and casual sexual partners, of which transmission between spouses accounts for approximately 20%–40% of cases [5], and is currently taken as one of the important aspects of sexual transmission into account in China [6, 7].

Although there have been recent developments in preventing sexual transmission of HIV, several HIV-negative spouses in HIV-serodiscordant couples were exposed to the high risk of HIV infection via sexual transmission due to inconsistent condom usage. A previous study revealed that the transmission rate in serodiscordant couples was 20–25/100 person-years for those who were unaware of their partner's HIV infection [8]. Similarly, a research conducted in Africa reported that the transmission rate in serodiscordant couples was significantly lower 5–10/100 person-years for those who were aware of their partner's HIV infection [9]. Thus, it is crucial to explore HIV-index cases as early as possible.

In recent years, although several scholars concentrated on the rate of HIV incidence in HIV-serodiscordant couples in China, however, the results were controversy. For instance, in China, the HIV/AIDS transmission rate of couples who were infected through blood transmission in Hebei province was 19%, with an incidence density of only 1.7/100 person-years [10]. However, the transmission rate was often significantly higher in other regions; for example, in Anhui province, it was around 30% [11], while in Yunnan and Guangxi provinces, it reached 42% and 60%, respectively [12–14].

A number of studies have reported HIV seroconversion rates among serodiscordant couples in different countries [9–16]. However, situation, cultural and research background, as well as patients' characteristics (e.g. antiretroviral therapy (ART)) were different, which resulted in large differences among various studies even those conducted in the same country [9–16]. Recently, the HIV/AIDS epidemic situation in Lu'an city (Anhui province, China) has remarkably increased. Our earlier studies showed that the HIV transmission rate among discordant couples was 30% in Lu'an, accompanying by a higher rate of male-to-female transmission rate compared with the female-to-male transmission rate [11]. However, with the increased understanding of HIV/AIDS prevention and treatment, we expect to observe a tangible change in the rate of HIV transmission and the relevant influencing factors. A limited number of research studies were conducted to examine various factors, covering HIV serodiscordant couples using longstanding and observational data. In this study, we analysed the HIV status of index cases in Lu'an using data collected during the period of 1999–2016, in order to study HIV transmission in serodiscordant couples. We herein aim to estimate HIV incidence among HIV discordant couples in Lu'an, and identify influencing factors associated with HIV transmission between couples.

## Subjects and methods

### Subjects

We have screened several HIV cases in different medical institutions at Lu'an since 1997. The first case of HIV infection was discovered in 1999, and thereafter, we continuously monitored the rate of HIV transmission in couples. Details of the serosurvey have been previously published [11]. In addition, a series of interventions, which included the promotion of condom usage, the elimination of unprotected sex, antiviral treatment, regular HIV testing and compliance education, were given to serodiscordant couples to reduce anally/vaginally HIV transmission rate during sexual intercourse [17–20].

**Fig. 1.**
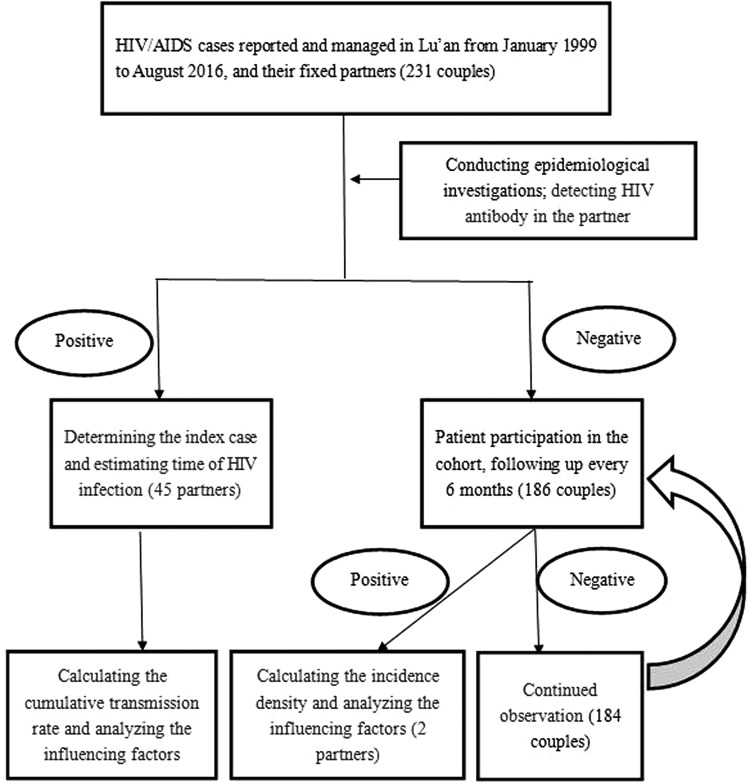
Flowchart of detection and assessment of HIV cases.

We followed-up the CDC data of patients with HIV/AIDS who had a fixed partner and lived in Lu'an during January 1999 to August 2016 (Fig. 1).

HIV transmission between couples refers to the existing HIV/AIDS patients who transmit the HIV during sexual intercourse, in which the first infected patient is known as an index case. HIV serodiscordant couples refer to couples that the infected patient has not transmitted the HIV to their partner.

Inclusion criteria were as follows: (1) discordant HIV infection couples, if one spouse was HIV-positive and the other was HIV-negative; or concordant HIV infection couples, if both spouses were HIV-positive, while one spouse was infected with HIV via sexual transmission by the other; (2) the fixed partner has no history pertaining to the risk of HIV infection, such as intravenous drug abuse, multiple sexual partners, blood transfusion, etc.; (3) the couples had a stable marriage and lived together for more than 6 months; (4) the age of couples was in the range of 18–70 years and (5) both spouses provided informed consent for the purposes of conducting research and publication of the results.

Exclusion criterion included either one or both spouses did not meet inclusion criteria.

### Survey method

#### The cohort

The eligible subjects were included in the cohort, from the date of diagnosis of the index case to follow-up the investigations ended on 31 December 2016. The dependent variable was seroconversion occurrence among HIV-negative partners during the follow-up period. Truncation variable was defined as either one or both spouses passed away during follow-up due to different reasons.

Blood samples belonged to HIV-positive patients analysed using enzyme-linked immunosorbent assay through a preliminary screening in several medical institutions were confirmed by western blotting in a confirmatory laboratory. HIV-positive patients were recorded in the national database. Subsequent epidemiological surveys were carried out and relevant information was collected. HIV antibodies were also detected in the fixed partners of infected patients. HIV concordant positive couples during the first screening were investigated to determine the course of transmission in spouses and the index case; one of the spouses had a history of paid blood donation (plasma) or blood donation before 1995, multiple sexual partners or drug abuse and the CD4 count was significantly lower than that of the other, while the other did not have the above risk factors. We independently established archives for HIV-negative spouses and included them in the cohort of serodiscordant couples, along with follow-up investigations every 6 months.

Determination of time of HIV infection was performed as the time of HIV infection from paid blood donors (slurry), that was mainly concentrated in 1995, and the time of HIV infection of the recipient, which was determined according to the suspected time of blood transfusion. The time of HIV infection from other sources was inferred based on epidemiological investigation and the CD4+ T lymphocyte count. According to the epidemiological survey, there is a clear time of occurrence of high-risk behaviours, such as prostitution and whoring, paid blood donation, drug abuse, etc., in which the time of occurrence refers to the time of infection. If the time of occurrence of dangerous behaviours is not enough clear, the CD4 count level can be used to estimate that time; if the number of CD4 cells is more than 500 cells, the time of HIV infection is within 3 years; if the CD4 count is equal to 350 cells, the time of HIV infection is around 5 years and if the CD4 count is less than 200 cells, the time of HIV infection is more than 7 years [21]. The cumulative transmission rate and the transmission density are calculated as follows:
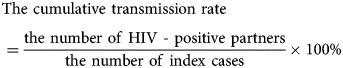

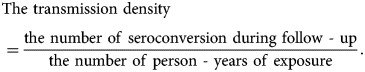


The period of exposure in person-years is the sum of the years that the index cases and their spouses have lived together. The interval from HIV infection to confirmation is the difference between HIV infection date and confirmation date, divided by 365.25. The end-point refers to the date in which the outcome occurs, or the date of ending the experiment for those who did not have the outcome.

To design our questionnaire, we used the questionnaire provided by Kong *et al*. [22], which was basically the same as ours on the purpose and main contents. The reliability test presented by Kong *et al*. [22] showed that Cronbach's *α* ranged from 0.88 to 0.93, and the validity test demonstrated that the cumulative variance contribution rate was 58.92%. In the current study, the questionnaire was designed based on the above-mentioned questionnaire, that associated with the actual situation and its contents, such as social demographic information, behavioural information, medical history and laboratory data of the patients.

### Quality control

The investigators who trained uniformly were all front-line professionals engaged in the treatment, management and relief of HIV patients. As they provided various medical assistance and life assistance services for HIV patients throughout the year, they have gained full trust and cooperation from the investigation subjects, and the information collected was accurate and reliable. All questionnaires were checked and qualified before double entry into the database.

### Statistical analysis

All data were imported into a pre-established database using Epidata 3.1 software. All statistical analyses were carried out using SPSS 10.01 software (SPSS Inc., Chicago, IL, USA) for calculation of mean, standard deviation (s.d.) and various rates. The *χ*^2^ test and multivariate logistic regression analysis were used for subsequent analysis. *P* < 0.05 was considered statistically significant.

### Ethical statement

All investigations and HIV tests were voluntarily carried out with patients' informed consent. All participants gave approval for their samples to be used for the purpose of controlling and preventing HIV. Written informed consent was obtained from all participants as well.

## Results

### Patients' clinical characteristics

A total of 231 HIV heterosexual serodiscordant couples were investigated, among whom 72.29% (167/231) of the index cases were male. Regarding the mean age of index cases, it was 41.86 ± 11.09 years with a range of 22–70 years for males, and 34.54 ± 10.01 years with a range of 19–62 years for females (*t* = 4.566, *P* < 0.001). To present the mean age of possible HIV infection for index cases, the mean age was 36.59 ± 10.75 years for males, and 28.46 ± 8.76 years for females (*t* = 5.846, *P* < 0.001). The interval from HIV infection to confirmation was as follows: it was 5.27 ± 3.44 years for males, and 6.08 ± 3.66 years for females (*t* = −1.568, *P* = 0.118). In addition, the mean age of the first sexual contact with spouse after being infected with HIV was 36.98 ± 10.56 years for males, and 29.44 ± 8.61 years for females (*t* = 5.097, *P* < 0.001).

### HIV transmission rate among couples

Because several households were largely failed to intervene prior to diagnosis of the index case, a total of 19.48% (45/231) households have already had HIV transmission between spouses at the time of detection of the first case in couples. The transmission rates between male-to-female and female-to-male spouses were 21.56% (36/167) and 14.07 (9/64), respectively (*χ*^2^ = 1.420, *P* = 0.233). Importantly, those 45 households who were concordant at the start of cohort should not be included in the transmission rate. Hence, subsequent analyses focused on the remaining 186 HIV serodiscordant couples, with a total of 507.7 person-years and a mean age of 2.73 years. A total of two couples (1.08%) were seroconverted to concordant HIV-positive, with a seroconversion rate of 0.39 per 100 person-years (2/507.7). The cumulative transmission rate of 1.08% (2/186) was significantly lower than 19.48% before intervention (*χ*^2^ = 35.714, *P* < 0.001). Moreover, in the absence of intervention, the HIV transmission rate between couples prior to diagnosis of the index case was roughly 23 times higher than that after diagnosis (RR = 22.753, 95.0% confidence interval (CI) 5.443–95.106). Two of the HIV negative partners who were seroconverted during the follow-up were male, aged 37 and 61 years, respectively, with follow-up duration of 7.1 and 4.5 years, respectively. The HIV transmission rates between male-to-female and female-to-male spouses were 0 (0/131) and 3.62 (2/55), respectively. Of the 186 HIV-discordant spouses, 119 (63.98%) HIV-positive partners received ART within 1 month after diagnosis. Among these couples, no seroconversions occurred during the follow-up. There were totally two seroconversions among the 67 couples (2.99%), in which the HIV-positive partners did not immediately receive antiviral treatment.

### Factors influencing HIV seroconversion

HIV incidence was the highest when the CD4 level was not detected at the end-point, while it was the lowest when the CD4 count was ⩾300 cells/mm^3^ (*χ*^2^ = 9.050, *P* = 0.029; Table 1). High HIV viral load at both the first visit and end-point was associated with an increased risk of transmission (*χ*^2^ = 17.714 and 12.944, respectively, *P* < 0.001 and 0.013, respectively; Table 1). HIV incidence was increased with age at the time of diagnosis, from 4.55% amongst those aged 18–25 years to 29.42% amongst those aged 45–70 years (*χ*^2^ = 7.947, *P* = 0.047; Table 1). High frequency of sexual activity prior to diagnosis was associated with an increased risk of transmission (*χ*^2^ = 8.719, *P* = 0.033; Table 1).

**Table 1.** Rates of HIV seroconversion and transmission among HIV serodiscordant couples: univariate analysis

Characteristic	Spouses	Transmission	Rate (%)	*χ*^2^	*P* value
Gender					
Male	167	36	21.56	0.545	0.460
Female	64	11	17.19		
Education level					
Primary school and below	69	11	15.94	1.765	0.623
Middle school	119	26	21.85		
High school	26	7	26.92		
College degree and above	17	3	17.65		
Route of infection					
Paid blood donors (slurry)	16	3	18.75	5.920^a^	0.205
Transfusion	37	7	18.92		
Heterosexual transmission	126	29	23.02		
Homosexual	51	7	13.73		
Intravenous drug	1	1	100.00		
CD4 counts at first visit (cells/μl)					
Not detected^a^	20	8	40.00	4.563	0.207
<150	85	16	18.82		
150+	58	10	17.24		
300+	68	13	19.12		
CD4 counts at the end (cells/μl)					
Not detected^a^	24	10	41.67	9.050	0.029
<150	41	11	27.50		
150+	48	8	16.67		
300+	118	18	15.25		
PVL at first visit (copies/ml)					
Not detected^a^	109	35	32.11	17.714	0.000
<300	97	9	9.28		
⩾300	25	3	12.00		
PVL at the end (copies/ml)					
Not detected^a^	92	29	31.52	12.944	0.002
<300	121	14	11.57		
⩾300	18	4	22.22		
Age confirmed^b^					
⩽25	22	1	4.55	7.947	0.047
25+	66	16	24.24		
35+	81	13	16.05		
45–70	62	17	29.42		
Age infected^c^					
⩽25	50	8	16.00	3.938	0.268
25+	79	16	20.25		
35+	72	13	18.06		
45–70	30	10	33.33		
Age of first sexual contact					
⩽25	44	7	15.91	3.689	0.297
25+	81	17	20.99		
35+	75	13	17.33		
45–70	31	10	32.26		
Sexual activity before diagnosis					
⩾3 times/week	35	8	22.86	8.719	0.033
1–2 times/week	70	19	27.14		
Semi-monthly	45	12	26.67		
Once a month	81	8	9.88		
Condom use					
Never used	169	38	22.49	5.108	0.164
Sometimes used	51	8	15.69		
Used frequently	3	1	33.33		
Used every time	8	0	0.00		
Period in time					
2006 and before	24	8	33.33	4.351	0.114
2007+	42	5	11.90		
2011+	165	34	20.61		

a Before 2010, there was no HIV confirmation laboratory, CD4 count and PVL test laboratory in Lu 'an. All the blood samples with HIV-positive screening need to be sent to the province HIV confirmation laboratory for confirmation experiment. HIV-positive patients were further tested for CD4 count and PVL. Many patients were tested for HIV at an advanced stage of AIDS and died before positive results were reported, failing to detect CD4 count and PVL further. The same below.

b Age at which HIV positive was first detected. The same below.

c Age at the time of HIV infection. The same below.

Variables with *P* < 0.05 in the above-mentioned univariate analysis were imported into the logistic regression model for multivariable analysis. The results showed that people in the age range of 18–25 years (Table 2) and with a HIV viral load of <300 copies/ml at the first visit (Table 2) were protective factors for HIV transmission, whereas a higher frequency of sexual activity is a significant risk factor, and the higher the frequency, the greater the risk (Table 2).

**Table 2.** Factors influencing HIV seroconversion among serodiscordant couples: multivariable analysis

Variable	RR	95.0% CI	*P* value
Age confirmed** (45–70 years as control)			
<25 years	0.059	0.006–0.553	0.013
25+ years	0.660	0.263–1.656	0.376
35+ years	0.542	0.216–1.357	0.191
Sexual activity before diagnosis (once a month as control)			
⩾3 times/week	6.780	1.949–23.591	0.003
1–2 times/week	5.841	2.158–15.807	0.001
Semi-monthly	3.358	1.186–9.510	0.023
PVL at first visit (not detected* as control)			
<300 copies/ml	0.163	0.069–0.387	<0.001
⩾300 copies/ml	0.256	0.065–1.012	0.052

### Factors influencing HIV seroconversion from male index cases

Univariate analysis showed that there was a strong association of HIV incidence with the CD4 level at the end point, HIV viral load at the first visit and end-point, age at the time of diagnosis and sexual activity before diagnosis (Table 3). Furthermore, the lower the CD4 level, the higher the HIV viral load at both the first visit and end-point. An older patient at the first diagnosis of the index case and higher frequency of sexual activity were associated with a higher HIV transmission rate. In multivariable analysis of risk factors for HIV retransmission to their HIV-negative partner, sexual activity with a frequency of 1–2 times/week resulted in an increase in HIV seroconversion, whereas the viral load of <300 copies/ml at the first visit led to a decrease (Table 4).

**Table 3.** Rates of HIV seroconversion and transmission among HIV serodiscordant couples: univariate analysis from male index cases

Characteristic	Spouses	Transmission	Rate (%)	*χ*^2^	*P* value
Education level					
Primary school and below	40	8	20.00	0.490	0.921
Middle school	87	19	21.84		
High school	23	6	26.09		
College degree and above	17	3	17.65		
Route of infection					
Paid blood donors (slurry)	5	1	20.00	7.311	0.120
Transfusion	21	3	14.29		
Heterosexual transmission	89	24	26.97		
Homosexual	51	7	13.73		
Intravenous drug	1	1	100.00		
CD4 counts at first visit (cells/μl)					
Not detected*	11	5	45.45	4.499	0.212
<150	70	15	21.43		
150+	39	9	23.08		
300+	47	7	14.89		
CD4 counts at the end (cells/μl)					
Not detected*	15	7	46.67	10.403	0.015
<150	36	11	30.56		
150+	37	8	21.62		
300+	79	10	12.66		
PVL at first visit (copies/ml)					
Not detected*	79	27	34.18	14.557	0.001
<300	70	7	10.00		
⩾300	18	2	11.11		
PVL at the end (copies/ml)					
Not detected*	66	21	31.82	8.736	0.013
<300	87	11	12.64		
⩾300	14	4	28.57		
Age confirmed**					
⩽25	9	0	0.00	9.102	0.028
25+	41	11	26.83		
35+	63	9	14.29		
45–70	54	16	29.63		
Age infected***					
⩽25	24	6	25.00	6.061	0.109
25+	54	10	18.52		
35+	63	10	15.87		
45–70	26	10	38.46		
Age of first sexual contact					
⩽25	20	5	25.00	5.046	0.168
25+	56	11	19.64		
35+	64	10	15.63		
45–70	27	10	37.04		
Sexual activity before diagnosis					
⩾3 times/week	20	4	20.00	11.066	0.011
1–2 times/week	42	15	35.71		
Semi-monthly	37	10	27.03		
Once a month	68	7	10.29		
Condom use					
Never used	118	27	22.88	3.766	0.288
Sometimes used	39	8	20.51		
Used frequently	3	1	33.33		
Used every time	7	0	0.00		
Period in time					
2006 and before	11	4	36.36	1.977	0.372
2007+	21	3	14.29		
2011+	135	29	21.48

**Table 4.** Factors influencing HIV seroconversion among serodiscordant couples: multivariable analysis from male index cases

Variable	RR	95.0% CI	*P* value
Sexual activity before diagnosis (once a month as control)			
⩾3 times/week	3.172	0.750–13.409	0.117
1–2 times/week	7.317	2.400–22.304	<0.001
Semi-monthly	2.929	0.965–8.897	0.058
PVL at first visit (not detected* as control)			
<300 copies/ml	0.150	0.055–0.407	<0.001
⩾300 copies/ml	0.294	0.057–1.516	0.144

### Factors influencing HIV seroconversion from female index cases

Univariate analysis showed that there was a poor association of HIV incidence with various characteristics of the index case (Table 5), as well as an increase in HIV incidence associated with an increase in viral load at the end-point. However, there were no independent risk factors screened in multivariable logistic regression analysis.

**Table 5.** Rates of HIV seroconversion and transmission among HIV serodiscordant couples: univariate analysis from female index cases

Characteristic	Spouses	Transmission	Rate (%)	*χ*^2^	*P* value
Education level					
Primary school and below	29	3	10.34	2.002	0.367
Middle school	32	7	21.88		
High school	3	1	33.33		
Route of infection					
Paid blood donors (slurry)	11	2	18.18	1.000	0.606
Transfusion	16	4	25.00		
Heterosexual transmission	37	5	13.51		
CD4 counts at first visit (cells/μl)					
Not detected*	9	3	33.33	6.965	0.073
<150	15	1	6.67		
150+	19	1	5.26		
300+	21	6	28.57		
CD4 counts at the end (cells/μl)					
Not detected*	9	3	33.33	7.696	0.053
<150	5	0	0.00		
150+	11	0	0.00		
300+	39	8	20.51		
PVL at first visit (copies/ml)					
Not detected*	30	8	26.67	3.937	0.140
<300	27	2	7.41		
⩾300	7	1	14.29		
PVL at the end (copies/ml)					
Not detected*	26	8	30.77	6.342	0.042
<300	34	3	8.82		
⩾300	4	0			
Age confirmed**					
⩽25	13	1	7.69	1.564	0.668
25+	25	5	20.00		
35+	18	4	22.22		
45–70	8	1	12.50		
Age infected***					
⩽25	26	2	7.69	5.619	0.132
25+	25	6	24.00		
35+	9	3	33.33		
45–70	4	0	0.00		
Age of first sexual contact					
⩽25	24	2	8.33	4.519	0.211
25+	25	6	24.00		
35+	11	3	27.27		
45–70	4	0	0.00		
Sexual activity before diagnosis					
⩾3 times/week	15	4	26.67	2.320	0.509
1–2 times/week	28	4	14.29		
Semi-monthly	8	2	25.00		
Once more than half a month	13	1	7.69		
Condom use					
Never used	51	11	21.57	5.550	0.062
Sometimes used	12	0	0.00		
Used every time	1	0	0.00		
Period in time					
2006 and before	13	4	30.00	2.442	0.295
2007+	21	2	9.52		
2011+	30	5	16.67		

## Discussion

The current survey found that the mean age for HIV diagnosis of female index cases was 28 years, which was significantly lower than that of males, at 37 years. This is often observed during the stage of marriage, and it is not only easy for the HIV-positive patients to transmit HIV to their partners through sexual contact, but also to their children through mother-to-child transmission via pregnancy.

According to our survey, couples who didn't know their own or their partners' HIV status before diagnosis had a cumulative HIV transmission rate of 19.48%, which was in agreement with the data of Hebei province [10], while being lower than our previous findings [11] and the data of Yunnan province [14]. These results suggest that recent developments in the detection and treatment of HIV/AIDS have permitted the timely intervention for HIV patients, and effectively controlled the spread of HIV within the family. The major differences in the rates of HIV transmission across different regions may be due to differences in sexual behaviour, social and family stability or varying behavioural and biological characteristics of the research subjects. Furthermore, developed regions often have a higher level of healthcare for improving detection and treatment of sexually transmitted diseases, which may result in a lower rate of transmission. Discordant HIV infection couples often had little understanding of the risks posed by HIV infection, including failure to use condoms during sexual intercourse, leading to long-term high risk of HIV transmission.

Previous studies have demonstrated that the probability of male-to-female transmission rate of HIV was higher than that of female-to-male, suggesting that the HIV titre in semen was higher than vaginal discharge [9, 11, 14, 23]. Moreover, because of the characteristics of the female's genital anatomy, there is a larger area of sexual contact in females compared with males, which may justify why women are more susceptible to be infected with HIV than men [24]. Similar results were found in northern India, where the HIV seroconversion rate in partners arising from male index cases was 2.90 per 100 person-years, being far higher than 0.95 per 100 person-years in partners, arising from female index cases [25]. A recent survey suggests that women often have less power in sexual relationships due to cultural, economic and social stature [26]. A cross-sectional survey from rural Uganda showed that females with less social power have an increased risk of HIV infection [27]. Our study revealed that there were no significant differences in transmission rates between male-to-female and female-to-male subjects prior to diagnosis of the index case. However, the number of male-to-female cases was 1.5 times higher than female-to-male cases. In particular, during the follow-up period following diagnosis of the index cases, seroconverted partners were exclusively male, which was inconsistent with the above-mentioned literature that suggested men were more prone to be impulsive and have lower protection awareness than women. Another study suggested that male HIV-negative partners often have to work, reflecting that they have fewer opportunities for intervention services, leading to higher rates of HIV seroconversion than women [28]. The exact reason for the discrepancy in the HIV transmission rate between males and females requires further investigation.

The HIV seroconversion rate in discordant HIV infection couples is of great importance in evaluating the effects of marital intervention. Our survey showed that in HIV-negative partners, the HIV seroconversion rate after diagnosis of the index case was significantly lower than that prior to diagnosis. This rate was also lower than previous reports [16, 18], which indicated that HIV intervention achieved remarkable effects. Additionally, seroconversion roughly occurred 4 years after diagnosis of the index case, while other studies conducted in China showed that over half of seroconversions occurred after 2.5 years following diagnosis of the index case [18]. The extended seroconversion time among HIV-negative partners achieved in the current study suggested that the intervention measures achieved positive results, and the HIV transmission between discordant couples were virtually suppressed. On the other hand, due to the prolonged follow-up management time for HIV/AIDS patients, there might be a reduction in HIV/AIDS prevention and awareness between discordant couples. Therefore, continuous strengthening of HIV/AIDS prevention and awareness via daily intervention in HIV-positive patients and their spouses is required.

Frequency of sexual intercourse with HIV-negative spouse is closely associated with the probability of HIV exposure and risk of HIV transmission [29]. A study has shown that the probability of HIV transmission during single unprotected sex was between 0.9% and 1.2% [30]. A case–control study showed that sexual frequency was an independent risk factor for sexually transmitted HIV among couples [31]. Cohort studies showed that the higher the frequency of sexual activity, the higher the HIV seroconversion rate among HIV-negative partners [11]. Our current study showed that the higher the frequency of sexual intercourse prior to HIV diagnosis of the index case, the higher the transmission rate of HIV, associating with a clear dose–response relationship. However, there was a poor association of HIV incidence with the frequency of sexual behaviour in female index cases, which may be associated with the lower probability of female-to-male rate of transmission, or a lower rate than estimated sample size of female index cases. After diagnosis of the index case, a variety of interventions, such as the promotion of condom use, the elimination of unprotected sex, antiviral treatment, etc. were taken, in which the frequency of unprotected sex was reduced. A study in Zambia showed that timely intervention following diagnosis of index might be resulted in a significant decreased frequency of unprotected sexual intercourse between serodiscordant couples, as well as a significant drop in the HIV seroconversion rate [19]. Our current study showed that following diagnosis of the index case, the HIV seroconversion rate among HIV-negative partners was 1/23 of pre-diagnosis as a result of various intervention measures, which was similar to results obtained from previous research [23]. A study conducted in Hebei province showed that timely HIV diagnosis resulted in an 80% decrease in the HIV transmission rate between spouses compared with patients who had delayed HIV diagnosis by 4–17 years after infection [20]. Therefore, early HIV detection not only can reduce the frequency of sexual intercourse between couples, but also allow early intervention in order to prevent the spread of HIV.

The diagnosis of HIV-infection and counselling provides complementary reductions in sexual risk behaviours among steady heterosexual serodiscordant couples, while the risk of transmission has not been fully eliminated [32]. Only the sustained use of ART to achieve a suppressed viral load in HIV-positive individuals is associated with zero or near-zero risk of sexual and vertical HIV transmissions. Studies identified as the only ones in which full viral suppression was confirmed in the index cases under ART showed a pooled heterosexual transmission rate of zero per 100 person-years (95% CI 0–0.05) [33–38].

The ART could significantly reduce the plasma viral load (PVL) of HIV patients to the levels lower than the detection limit within 6 months of initial treatment, in addition to reduce semen and intracervical viral load to the lower limit [39]. The 052 research project on AIDS conducted in 2011 revealed that early initiation of ART resulted in a 96% reduction in the number of HIV-1 linked transmissions compared with delayed therapy, as well as a 41% reduction in the number of HIV-1-related clinical events [40]. Studies performed in China have shown that ART could effectively block the spread of HIV between spouses in discordant couples [23, 41]. A meta-analysis showed that ART could reduce the risk of HIV transmission in middle- and high-income countries by 70% and 30%, respectively [41]. A longitudinal study performed in Uganda showed that the HIV seroconversion rate in HIV-positive partners who did not receive ART was 7.35/100 person-years, compared with 0 case for those who received ART [15]. The current study also showed that regarding the two cases who did not receive ART due to the non-compliance with the antiviral treatment standard, both cases resulted in seroconversion in their partners. In contrast, no seroconversion occurred among the 119-negative spouses whose partners received ART. Therefore, a prompt intervention in HIV discordant couples is required in order to reduce the risk of HIV transmission.

CD4+ T lymphocyte count provides an appropriate reflection of the patient's immune status and HIV activity. Lower CD4 counts indicates worsened immunity, and the more active the virus, the greater likelihood of HIV transmission among spouses. A previous study showed that the viral load in the semen of HIV-positive patients was significantly lower when the CD4+ T lymphocyte count was greater than 350 cells/μl [42]. Furthermore, index cases with CD4+ T lymphocyte count of less than 350 cells/μl resulted in 5.7 times greater risk of HIV seroconversion among HIV-negative partners in China [29]. In the current study, there was no statistical correlation between CD4+ T lymphocyte count and the rate of HIV transmission among spouses, which is mainly due to the fact that the majority of HIV transmission between spouses had occurred roughly 5 years prior to diagnosis of index case, and therefore there would be a disparity in the level of CD4+ T lymphocyte count at the time of diagnosis.

At present, there are no studies, which link age with HIV susceptibility. However, a study showed that index cases at different age groups had different HIV transmission rates [11]. In this study, diagnosis of index cases in patients under the age of 25 years old had the lowest HIV seroconversion rate, which might be related to the following reasons: (i) people under the age of 25 years old had greater awareness of HIV testing, allowing timely detection of HIV infection, thereby reducing their exposure; (ii) at the age of marriage, there were more HIV testing opportunities, such as pre-pregnancy and prenatal examinations and (iii) the majority of individuals in this age group had to work, demonstrating that the partners often lived separately and had an unstable lifestyle, thereby reducing any opportunities for exposure.

Numerous studies have confirmed the relationship between viral load in HIV-positive blood and probability of sexual transmission. Higher PVL and viral load in semen and vaginal secretions are directly associated with the risk of HIV transmission to uninfected partners through sexual contact [42, 43]. A study in Uganda showed that when plasma HIV-1 level of the index cases was <1500 copies/ml for 2.5 years, there was no sexual transmission of HIV in serodiscordant couples [44]. Our current study found that higher viral load was a risk factor for HIV transmission in both male and female index cases. Furthermore, PVL >300 copies/ml was shown to be a risk factor in male index cases, whereas PVL <300 copies/ml was a protective factor in female index cases.

Improper or lack of condom usage during sex is often a direct cause of HIV transmission among discordant couples. Studies have shown that adherence to condoms is a strong protective factor for HIV transmission in discordant couples [18, 28]. Appropriate usage of condom during sex could reduce the risk of sexually transmitted HIV by 90% [45]. We postulated that couples who frequently used condom also had a higher frequency of sexual activity compared with couples who occasionally or never used condom. Our epidemiological studies of two spouses who seroconverted during the follow-up period revealed that they had a higher frequency of marital sex, even though they frequently used condom, they failed to routinely use condom. Only systematic use of condom has preventive efficacy. Several meta-analyses of observational and cohort studies have found that 100% use of condom can reduce HIV transmission in heterosexual couples by around 80% [46]. It also could be attributed to a relatively small number of cases of discordant couples who used condom prior to diagnosis of the index case, which might be attributed to the unpredictability of the results.

Our current study found that there were several factors, which influenced the transmission rate of HIV between male and female index cases. In male index cases, CD4+ T lymphocyte count, PVL, frequency of sexual activity and age of diagnosis were factors affected the rate of HIV transmission to the spouse. In contrast, in female index cases, PVL was the only factor affected the rate of HIV transmission to the spouse. However, this result might be slightly skewed due to the relatively small sample size of female index cases. The specific reasons for this trend require further investigations.

This study provided reliable evidence for various factors, involving HIV serodiscordant couples based on longstanding study, in which relevant research data were rarely reported in China. In this study, we found the reduction of HIV transmission rate after the diagnosis of the index case, in addition to the low or null risk of transmission to index cases with antiviral treatment, indicating that the current policy of expanded testing and expanded ART for all HIV patients in China can be confirmed and should be maintained as well. However, there are several limitations in our study. The majority of survey's information involved personal privacy, and it is also possible that respondents might deliberately suppress or withhold their answers, which might lead to information bias. The HIV transmission relationship among concordant HIV-positive couples was determined based on epidemiological information, which might result in possible bias due to the lack of subtype analysis and molecular biological evidence. The total number of female index cases was relatively small, which might have resulted in slight inaccuracies in the findings.

## Conclusions

HIV transmission rate among serodiscordant couples was high, and there was no statistical difference in the transmission rate between male-to-female and female-to-male spouses prior to diagnosis of the index case. However, following diagnosis, the transmission rate was significantly reduced, especially the risk of transmission in the index case with antiviral treatment was null, and the rate of female-to-male transmission was higher than that of male-to-female. Therefore, a prompt intervention in HIV discordant couples with ART of index case is essential to reduce the risk of HIV transmission.
